# Exploring the impact of deubiquitination on melanoma prognosis through single-cell RNA sequencing

**DOI:** 10.3389/fgene.2024.1509049

**Published:** 2024-12-05

**Authors:** Su Peng, Jiaheng Xie, Xiaohu He

**Affiliations:** ^1^ Department of Plastic Surgery, The Affiliated Friendship Plastic Surgery Hospital of Nanjing Medical University, Nanjing, China; ^2^ Department of Plastic Surgery, Xiangya Hospital, Central South University, Changsha, China

**Keywords:** melanoma, prognostic model, deubiquitination, single-cell RNA sequencing, immune microenvironment

## Abstract

**Background:**

Cutaneous melanoma, characterized by the malignant proliferation of melanocytes, exhibits high invasiveness and metastatic potential. Thus, identifying novel prognostic biomarkers and therapeutic targets is essential.

**Methods:**

We utilized single-cell RNA sequencing data (GSE215120) from the Gene Expression Omnibus (GEO) database, preprocessing it with the Seurat package. Dimensionality reduction and clustering were executed through Principal Component Analysis (PCA) and Uniform Manifold Approximation and Projection (UMAP). Cell types were annotated based on known marker genes, and the AUCell algorithm assessed the enrichment of deubiquitination-related genes. Cells were categorized into DUB_high and DUB_low groups based on AUCell scores, followed by differential expression analysis. Importantly, we constructed a robust prognostic model utilizing various genes, which was evaluated in the TCGA cohort and an external validation cohort.

**Results:**

Our prognostic model, developed using Random Survival Forest (RSF) and Ridge Regression methods, demonstrated excellent predictive performance, evidenced by high C-index and AUC values across multiple cohorts. Furthermore, analyses of immune cell infiltration and tumor microenvironment scores revealed significant differences in immune cell distribution and microenvironment characteristics between high-risk and low-risk groups. Functional experiments indicated that TBC1D16 significantly impacts the migration and proliferation of melanoma cells.

**Conclusion:**

This study highlights the critical role of deubiquitination in melanoma and presents a novel prognostic model that effectively stratifies patient risk. The model’s strong predictive ability enhances clinical decision-making and provides a framework for future studies on the therapeutic potential of deubiquitination mechanisms in melanoma progression. Further validation and exploration of this model’s applicability in clinical settings are warranted.

## Introduction

Cutaneous melanoma is a malignant tumor characterized by the abnormal proliferation of melanocytes, the cells responsible for producing skin pigment (Long et al., 2023; Arnold et al., 2022; DE Gruijl and Armstrong, 2022). This type of cancer is known for its high aggressiveness and metastatic potential, typically presenting on the skin’s surface but also occurring in other areas, such as the eyes and internal organs (Bertolotto, 2021; Kostaki et al., 2023; Buja et al., 2022). Over the past few decades, the incidence of melanoma has significantly increased, particularly among white populations, and is closely associated with ultraviolet (UV) exposure, such as that from sunlight (Lopes et al., 2021). Early diagnosis and treatment are crucial for improving patient prognosis; however, late-stage melanoma patients often experience low survival rates and limited treatment options (Carpi et al., 2020; Garbe et al., 2022; Orzan et al., 2015).

The clinical manifestations of melanoma are diverse, with common features including irregular moles or skin lesions, uneven coloration, indistinct borders, and an increase in diameter (Sergi et al., 2023; Ribeiro Moura Brasil Arnaut et al., 2021; Everett et al., 2019). Recent advancements in immunotherapy and targeted therapies have provided new treatment options for melanoma patients, although responses can vary due to individual differences, leading some patients to respond poorly to existing treatments (Leonardi et al., 2020; Hoeijmakers et al., 2023; Xiao et al., 2024). Consequently, the exploration of novel biomarkers and therapeutic targets has become a focal point of current research.

Ubiquitination is a critical post-translational modification process in which a small protein, ubiquitin, is attached to target proteins (Popovic et al., 2014). This process typically occurs through a three-step reaction: first, ubiquitin is activated by E1 enzymes (ubiquitin-activating enzymes) and transferred to E2 enzymes (ubiquitin-conjugating enzymes); subsequently, E3 enzymes (ubiquitin ligases) transfer ubiquitin to the target protein (Cockram et al., 2021; Mansour, 2018; Liu et al., 2024). The addition of ubiquitin generally marks the target protein for degradation, playing a key role in the quality control of intracellular proteins and signal transduction (Ye et al., 2023). Deubiquitination is the counter-process to ubiquitination, mediated by deubiquitinating enzymes (DUBs), which are responsible for removing ubiquitin molecules from target proteins (Jiang et al., 2023; Wu et al., 2020). This process not only affects protein stability and degradation but also has far-reaching implications for key biological processes, including signal transduction, cell proliferation, and apoptosis (Hu et al., 2021). Dysregulation of DUB activity is associated with the progression of various cancers, influencing how cells respond to environmental stimuli and their sensitivity to treatments (Han et al., 2022).

In recent years, the role of deubiquitination in melanoma progression has garnered increasing attention. Numerous studies have demonstrated that deubiquitinating enzymes (DUBs) in melanoma can influence cell proliferation, apoptosis, and metastatic potential by modulating the stability of tumor suppressor genes and oncogenes. For example, Feng et al. discovered that USP11 promotes melanoma progression by stabilizing the protein level of NONO through deubiquitination (Feng et al., 2021). Furthermore, targeting the USP7/RRM2 axis induces senescence, sensitizing melanoma cells to HDAC/LSD1 inhibitors (Granieri et al., 2022). Consequently, targeting the deubiquitination process is considered a promising approach for developing new therapies against melanoma. Therefore, a deeper understanding of the mechanisms of ubiquitination and deubiquitination is essential for elucidating the biological characteristics of melanoma and developing effective therapeutic strategies.

## Methods

### Data acquisition and preprocessing

Single-cell RNA sequencing data for cutaneous melanoma were obtained from the Gene Expression Omnibus (GEO) under accession number GSE215120 (Zhang et al., 2022; Zhang et al., 2024). Specifically, we extracted three datasets (GSM6622299, GSM6622300, and GSM6622301), each representing samples from cutaneous melanoma. The raw count matrices were downloaded and further processed using the Seurat package in R. Cells with fewer than 200 expressed genes or with over 10% mitochondrial content were excluded from subsequent analyses to ensure data quality. In addition to single-cell RNA sequencing data, bulk RNA sequencing data were downloaded from The Cancer Genome Atlas (TCGA) and four additional GEO datasets: GSE19234, GSE22153, GSE59455, and GSE65904 (Bogunovic et al., 2009; Jönsson et al., 2010; Budden et al., 2016; Cabrita et al., 2020). These datasets were selected to provide a comprehensive view of gene expression across larger patient cohorts. TCGA data were accessed via the Genomic Data Commons (GDC) portal, while the GEO datasets were obtained directly from the GEO database. Each dataset was processed uniformly to ensure consistency. Briefly, raw count data were normalized and log-transformed. For each sample, genes with low expression levels were filtered out based on a minimum threshold to remove noise. We also used batch correction methods to minimize technical variation between the datasets.

### Dimensionality reduction and clustering

Following normalization and scaling of the data, we performed principal component analysis (PCA) for dimensionality reduction. The top 20 principal components were selected based on the Elbow plot and subjected to Uniform Manifold Approximation and Projection (UMAP) to visualize the data in a two-dimensional space. Clustering was conducted using the Louvain algorithm with a resolution parameter of 0.5, identifying distinct cellular populations within the melanoma samples.

### Cell type annotation

Cluster annotation was performed based on the expression of known marker genes. These annotations were manually curated to ensure accurate classification of various cell types.

### AUCell analysis for deubiquitination gene enrichment

To assess the activity of deubiquitination-related genes across different cell populations, we applied the AUCell algorithm. A predefined gene set associated with deubiquitination was used, and AUCell scores were calculated for each cell, providing a measure of gene set enrichment. The resulting scores were visualized across clusters to identify potential differences in deubiquitination activity between cell types.

### Identification of DUB_high and DUB_low groups

For determining the AUCell score threshold, we grouped cells into high-activity and low-activity groups based on the median score value. Using the median as a threshold allowed us to effectively capture relative differences in deubiquitination activity across cell populations, reducing the influence of outliers on our analysis. We selected AUCell due to its suitability for single-cell RNA-seq data, as it calculates enrichment at the single-cell level and effectively handles variability in expression levels and data sparsity. The AUCell scores were visualized across clusters to identify cell-type-specific patterns of deubiquitination activity. Differential expression analysis was performed between these two groups using the FindMarkers function in Seurat. Genes with an adjusted p-value < 0.05 and |log2 fold change| > 0.25 were considered differentially expressed.

### Correlation and gene ranking

Additionally, the top 150 genes most correlated with the AUCell scores were identified by computing Spearman’s correlation coefficients between gene expression levels and AUCell scores. These genes were ranked based on their correlation coefficients for further analysis.

### Cell-cell communication analysis

To explore potential interactions between different cell types in DUB_high and DUB_low groups, cell-cell communication analysis was performed using the CellChat package. This analysis helped identify key signaling pathways and ligand-receptor interactions that may be altered between the two groups, providing insights into how deubiquitination activity impacts the tumor microenvironment and intercellular communication.

### Construction of a prognostic model using ensemble machine learning algorithms

First, we intersected the differentially expressed genes (DEGs) identified from single-cell sequencing analysis with the top 150 genes correlated with AUCell scores to derive key genes. In the TCGA cohort, we initially used the survival R package to screen for genes with prognostic significance. These candidate genes were then subjected to further filtration using univariate Cox regression analysis to assess their prognostic value. The resulting significant genes were used for the construction of the prognostic model.

The TCGA cohort was designated as the training set, while external cohorts (GSE19234, GSE22153, GSE59455, and GSE65904) served as validation sets. To enhance the robustness of the model, we employed 10-fold cross-validation and applied 101 combinations of 10 machine learning algorithms, including Stepwise Cox regression (StepCox), Lasso, Ridge, partial least squares regression for Cox (plsRcox), CoxBoost, Random Survival Forest (RSF), Gradient Boosting Model (GBM), Elastic Net (Enet), Supervised Principal Components (SuperPC), and Survival Support Vector Machine (survival-SVM). The objective was to identify the most valuable prognostic feature, referred to as the Prognostic Index Score (PIS), characterized by the highest concordance index (C-index). The model with the highest C-index in the validation sets was selected as the optimal deubiquitination-related signature (DRS).

### Model evaluation

After selecting the optimal Random Survival Forest (RSF) + Ridge prognostic model, we evaluated its performance across multiple cohorts, including the TCGA cohort (training set) and four external validation cohorts: GSE19234, GSE22153, GSE59455, and GSE65904.

Kaplan-Meier survival curves were constructed to compare overall survival (OS) between high-risk and low-risk groups, stratified based on the median Prognostic Index Score (PIS) in each cohort. Statistical significance was assessed using the log-rank test, with p-values <0.05 considered significant. Hazard ratios (HR) and 95% confidence intervals (CI) were calculated using Cox proportional hazards regression models to further quantify the association between risk scores and patient survival outcomes.

To assess the discriminative power of the RSF + Ridge model, time-dependent Receiver Operating Characteristic (ROC) curves were plotted for 1-, 3-, and 5-year overall survival in each cohort. The area under the ROC curve (AUC) was calculated to measure the predictive accuracy of the model, with higher AUC values indicating better prognostic performance. The timeROC R package was used for ROC curve generation and AUC calculations.

Principal component analysis (PCA) was performed to visualize the distribution of patients in the high-risk and low-risk groups based on their gene expression profiles. The top principal components were calculated using the expression of genes included in the RSF + Ridge model. PCA plots were generated to show how well the model separated the two risk groups, with distinct clustering indicating good separation. This analysis was conducted using the prcomp function in R, and the resulting plots were visualized using the ggplot2 package.

By applying survival analysis, ROC curves, and PCA, we rigorously assessed the robustness and generalizability of the RSF + Ridge prognostic model across multiple independent cohorts.

### Immune cell infiltration and tumor microenvironment score analysis

To investigate differences in immune cell infiltration and the tumor microenvironment (TME) between the high-risk and low-risk groups defined by the RSF + Ridge prognostic model, we employed several computational tools.

Immune cell infiltration was quantified using multiple immune deconvolution algorithms, including CIBERSORT, xCell, and MCP-counter, to estimate the relative abundance of immune cell types in each tumor sample. We obtained immune cell infiltration scores for various immune cell populations, such as CD8^+^ T cells, macrophages, NK cells, and B cells. The CIBERSORT algorithm was executed using the immune signature matrix, with a threshold of *p* < 0.05 for significant deconvolution results. Differences in immune cell infiltration between the high-risk and low-risk groups were compared using the Wilcoxon rank-sum test, with p-values < 0.05 considered statistically significant.

The ESTIMATE algorithm was used to calculate stromal and immune scores, which represent the presence of stromal and immune components in the tumor microenvironment. These scores were then combined to derive the ESTIMATE score, an overall measure of TME composition. We compared the stromal, immune, and ESTIMATE scores between the high-risk and low-risk groups using the Wilcoxon rank-sum test to identify significant differences.

To further explore the relationship between the Prognostic Index Score and immune infiltration or TME scores, Spearman’s correlation analysis was conducted. Correlation coefficients were calculated between the PIS and immune cell infiltration levels or TME scores, with p-values < 0.05 indicating significant associations.

### Gene knockdown and functional assays in A375 cell line

To explore the functional role of TBC1D16, a key gene identified in the RSF + Ridge prognostic model, we performed gene knockdown experiments in the A375 melanoma cell line. TBC1D16 was silenced using shRNA specific to TBC1D16 (sh-TBC1D16). A sh-control was used as the negative control.

### Colony formation assay

To assess the impact of TBC1D16 knockdown on the long-term proliferative capacity of A375 cells, a colony formation assay was performed. Briefly, 500 transfected A375 cells (shTBC1D16 and shControl) were seeded into 6-well plates and cultured in complete medium for 10–14 days, allowing colonies to form. The medium was replaced every 3 days. At the end of the incubation period, colonies were fixed with 4% paraformaldehyde for 15 min, stained with 0.1% crystal violet solution, and then washed with distilled water. The number of colonies containing at least 50 cells was counted manually under a light microscope. Colony formation efficiency was compared between sh-TBC1D16 and sh-Control groups using a two-tailed Student’s t-test, with *p* < 0.05 considered significant.

### Transwell migration assays

To evaluate the effects of TBC1D16 knockdown on cell migration, Transwell assays were conducted using 24-well Transwell chambers (Corning, 8-μm pore size). For the migration assay, 1 × 10^5^ transfected A375 cells (shTBC1D16 and shControl) were suspended in 200 µL serum-free medium and seeded in the upper chamber of the Transwell insert. The lower chamber was filled with 600 µL of medium containing 10% fetal bovine serum (FBS) as a chemoattractant. Cells were allowed to migrate for 24 h at 37°C in a humidified incubator. After incubation, non-migrated cells on the upper side of the membrane were removed with a cotton swab, and the cells that had migrated to the lower surface were fixed with 4% paraformaldehyde and stained with 0.1% crystal violet.

### Statistical analysis

All experiments were performed in triplicate, and data are presented as mean ± standard deviation (SD). Comparisons between two groups (shTBC1D16 vs. shControl) were conducted using a two-tailed Student’s t-test, with *p* < 0.05 considered statistically significant. For survival analysis, Kaplan-Meier curves with log-rank tests were applied, and hazard ratios (HR) were calculated using Cox regression. Receiver Operating Characteristic (ROC) curves and the area under the curve (AUC) were used to evaluate model performance. All analyses were performed using GraphPad Prism and R software.

## Results

### Single-cell RNA sequencing analysis of cutaneous melanoma microenvironment

**FIGURE 1:**
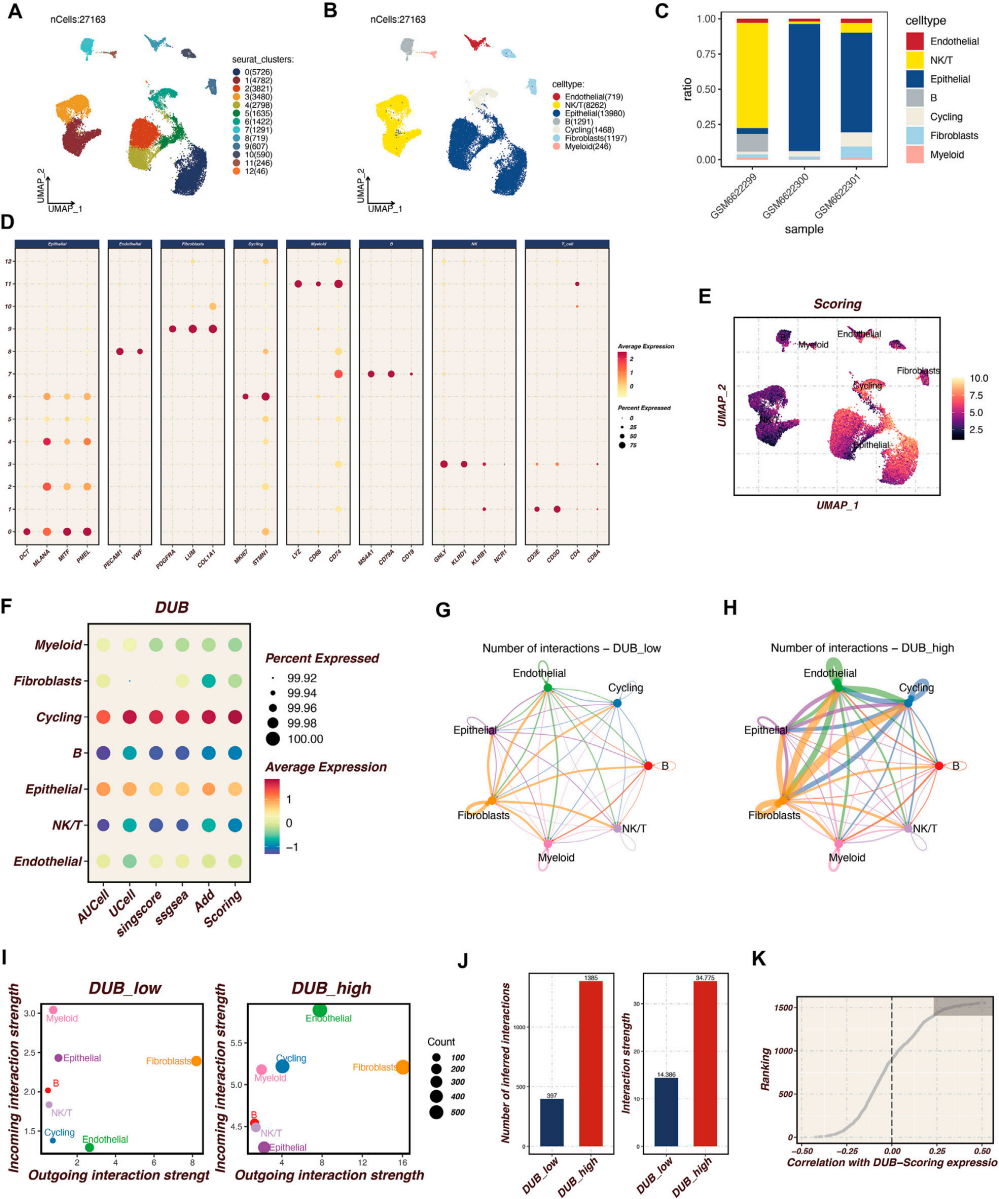
Single-Cell RNA Sequencing Analysis of Cutaneous Melanoma Microenvironment Single-cell RNA sequencing was conducted on cutaneous melanoma samples (GSM6622299, GSM6622300, and GSM6622301) to investigate the microenvironmental landscape. **(A, B)** The UMAP plot displays the clustering of 27,163 individual cells into distinct populations, identified through Seurat clustering methods and annotated for cell types. These populations comprise endothelial cells, NKT cells, epithelial cells, B cells, cycling cells, fibroblasts, and myeloid cells. **(C)** The proportional distribution of these cell types across the three samples highlights variability in cellular composition. **(D)** The expression patterns of selected marker genes across various cell types are shown, with dot size indicating the percentage of expressing cells and color intensity representing average expression levels. This confirms the successful identification of diverse cell populations. **(E)** AUCell scoring identifies cells exhibiting high activity of deubiquitination (DUB)-related genes, color-coded by DUB activity scores, with notable activity in cycling and epithelial cell populations. **(F)** The average expression levels and prevalence of DUB-related gene expression across cell types emphasize significant expression in cycling and epithelial cells. **(G, H)** Cell-cell communication analysis, conducted using the CellChat tool, compares interaction networks between DUB*low and DUB*high groups. The DUB_high cells, particularly in endothelial and fibroblast populations, demonstrate more extensive and stronger intercellular communication activity. **(I, J)** Specific quantitative analysis of intercellular communication. **(K)** Acquisition of the top 150 relevant genes.

Single-cell RNA sequencing analysis was performed on cutaneous melanoma samples (GSM6622299, GSM6622300, and GSM6622301), as illustrated in Figure 1. The UMAP plots in Figures 1A, B display the clustering of 27,163 cells into distinct cell populations based on Seurat clustering and cell type annotations. These populations include endothelial cells, NKT cells, epithelial cells, B cells, cycling cells, fibroblasts, and myeloid cells. Figure 1C shows the proportional distribution of these cell types across the three melanoma samples, highlighting the variability in cell type composition. Figure 1D depicts the expression patterns of selected marker genes across different cell types, with dot size representing the percentage of cells expressing each gene and color intensity reflecting the average expression level. This confirms the successful identification and annotation of diverse cell populations. In Figure 1E, AUCell scoring was employed to identify cells with high activity of deubiquitination (DUB)-related genes, coloring the cells according to their DUB activity scores. The highest scores were predominantly observed in cycling and epithelial cell populations. Figure 1F illustrates the average expression levels and percentages of cells expressing DUB-related genes across various cell types, with significant expression noted in cycling and epithelial cells. Figures 1G, H present the cell-cell communication analysis conducted using the CellChat tool, comparing interaction networks between the DUB_low and DUB_high groups. DUB_high cells, particularly those in the endothelial and fibroblast populations, exhibited more extensive and stronger intercellular communication compared to DUB_low cells. Figure 1I further quantifies the incoming and outgoing interaction strengths in the DUB_low and DUB_high groups, with the DUB_high group displaying significantly greater interaction strength, as shown in Figure 1J. The number of inferred interactions and interaction strength in the DUB_high group was higher and statistically significant. Finally, Figure 1K shows the correlation between DUB-related scoring and gene expression ranking, identifying the top 150 genes most associated with DUB activity. This correlation analysis reinforces the functional importance of DUB genes in driving cell interactions and activity within the tumor microenvironment. This analysis emphasizes the heterogeneity of the tumor microenvironment in cutaneous melanoma and highlights the enhanced communication networks driven by DUB_high cells.

### Identification of the optimal prognostic model

Our analysis revealed that the integrated machine learning approach successfully identified a set of prognostic genes from the intersection of differentially expressed genes and the 150 key genes derived from the single-cell sequencing data. Upon applying univariate Cox regression analysis within the TCGA cohort, we discerned several genes with significant prognostic value.

**FIGURE 2:**
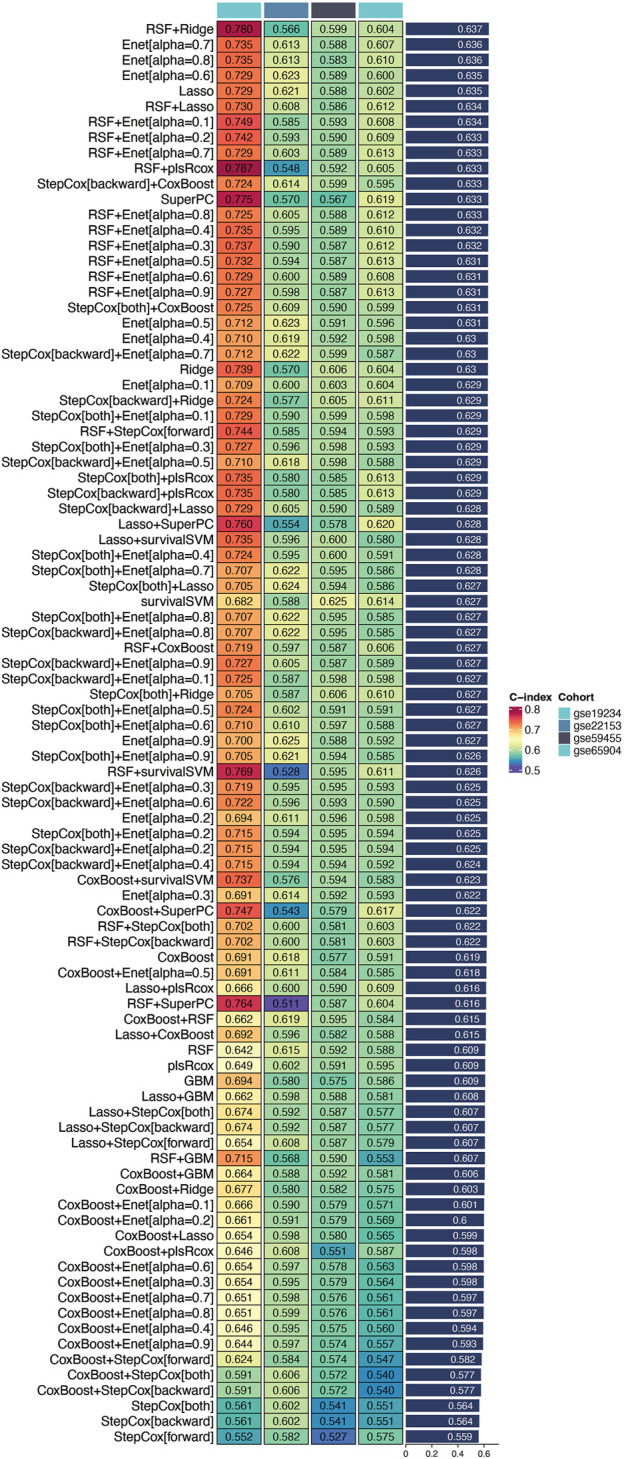
Identification of the optimal prognostic model. This figure illustrates the process of identifying a set of prognostic genes through an integrated machine learning approach. Differentially expressed genes were combined with the 150 key genes derived from single-cell sequencing data. Univariate Cox regression analysis applied to the TCGA cohort revealed several genes with significant prognostic value. Among 101 tested combinations, the Random Survival Forest (RSF) combined with Ridge regression was identified as the optimal model, achieving the highest concordance index (c-index) in the validation cohort. This model is designated as the DUB-related signature (DRS), indicating its potential as a robust prognostic tool for cutaneous melanoma.

Utilizing the TCGA cohort as the training set and validating against multiple datasets (GSE19234, GSE22153, GSE59455, and GSE65904), we conducted extensive cross-validation using various machine learning algorithms. Among the 101 combinations tested, the Random Survival Forest (RSF) combined with Ridge regression emerged as the optimal model, achieving the highest concordance index (c-index) in the validation cohort (Figure 2). This model was designated as the DUB-related signature (DRS), highlighting its potential as a robust prognostic tool for cutaneous melanoma. These findings underscore the efficacy of machine learning techniques in enhancing prognostic predictions and the relevance of the identified DUB-related signature in the context of melanoma prognosis.

### Survival analysis and model validation in multiple cohorts

**FIGURE 3:**
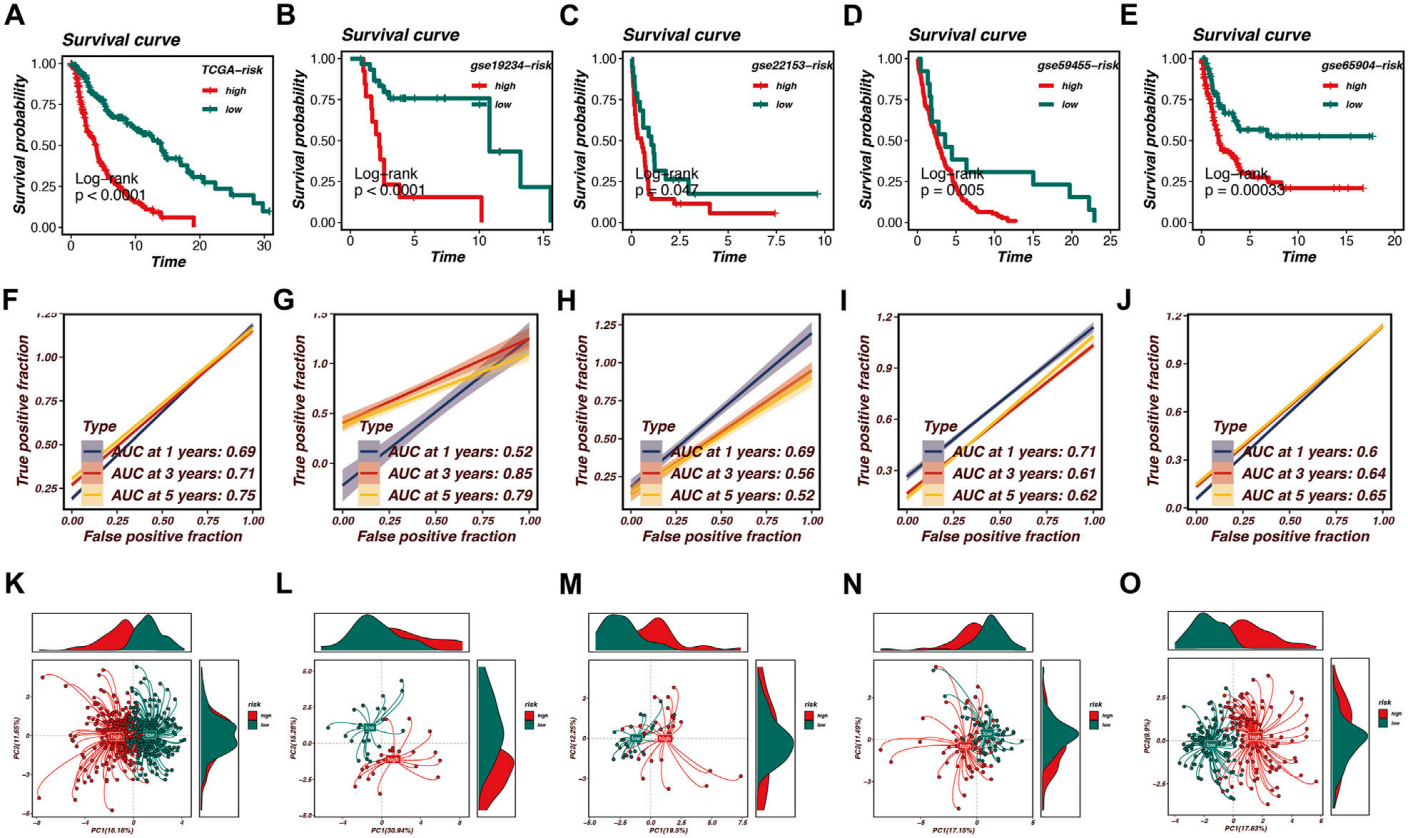
Survival analysis and model validation in multiple cohorts. Survival analysis across multiple datasets, including TCGA, GSE19234, GSE22153, GSE59455, and GSE65904, is shown in this figure. **(A–E)** Survival curves demonstrate significant differences in survival probabilities between high-risk and low-risk groups, with p-values consistently below 0.05, confirming the robustness of the model. **(F–J)** Receiver Operating Characteristic (ROC) curves illustrate the Area Under the Curve (AUC) values at various time points, reflecting the model’s accuracy in distinguishing between high- and low-risk patients. AUC values range from 0.52 to 0.85, with the TCGA cohort achieving an AUC of 0.75 at 5 years. **(K–O)** Principal Component Analysis (PCA) effectively segregates high-risk and low-risk patient groups based on the first two principal components. Density plots highlight the distribution of risk scores within the identified clusters, reinforcing the prognostic model’s effectiveness in predicting survival outcomes and capturing biological heterogeneity within melanoma samples.

After establishing the optimal prognostic model using Random Survival Forest (RSF) combined with Ridge regression, we performed survival analysis across multiple datasets, including TCGA, GSE19234, GSE22153, GSE59455, and GSE65904. The survival curves presented in Figures 3A–E indicate significant differences in survival probabilities between high- and low-risk groups across all cohorts, with p-values consistently below 0.05, confirming the robustness of our model in predicting patient outcomes.

Furthermore, we evaluated the predictive performance of the model using Receiver Operating Characteristic (ROC) curves. Figures 3F–J demonstrate the Area Under the Curve (AUC) values at various time points, reflecting the model’s accuracy in distinguishing between high- and low-risk patients. Notably, the AUC values ranged from 0.52 to 0.85 across the cohorts, with the TCGA cohort achieving an AUC of 0.75 at the 5-year mark, indicating strong predictive capability.

To further validate our model, Principal Component Analysis (PCA) was conducted, as illustrated in Figures 3K–O. The PCA plots effectively segregated high-risk and low-risk patient groups based on the first two principal components. This segregation is further highlighted by the density plots, which depict the distribution of risk scores within the identified clusters. The PCA analysis confirms that the prognostic model is not only effective in predicting survival outcomes but also in capturing the underlying biological heterogeneity within the melanoma samples.

Overall, the survival analysis, ROC curves, and PCA results underscore the validity of the RSF + Ridge model as a powerful prognostic tool in melanoma research, demonstrating its potential for clinical application in stratifying patient risk.

### Immune infiltration and tumor microenvironment analysis

**FIGURE 4:**
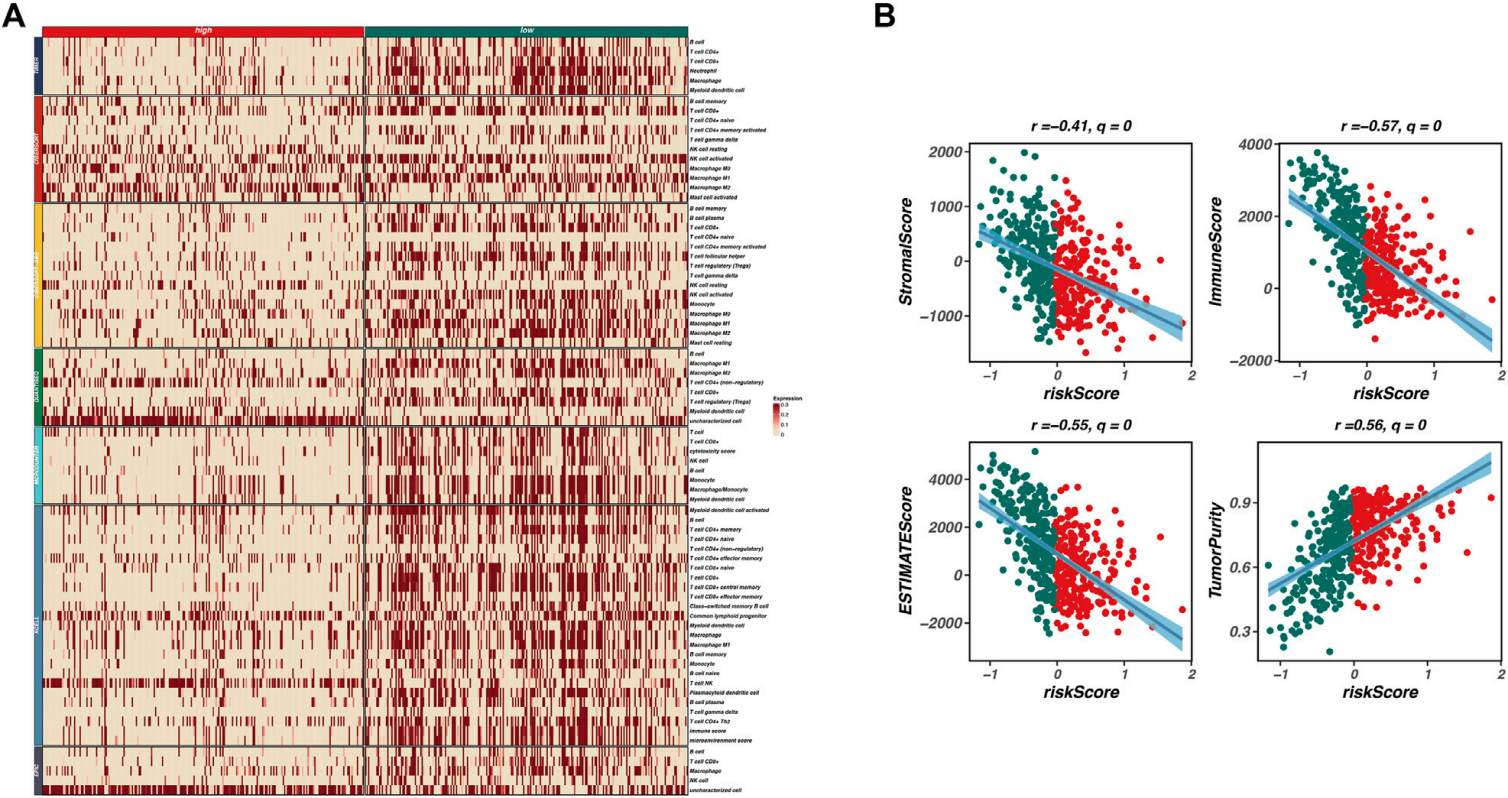
Immune infiltration and tumor microenvironment analysis. This figure investigates the relationship between the prognostic model and the tumor microenvironment (TME) through immune infiltration analysis. **(A)** A heatmap displays the expression levels of various immune cell types across high-risk and low-risk groups, revealing a distinct immune landscape with significant differences. High-risk patients exhibit a lower abundance of immune cell types, including CD4^+^ T cells, regulatory T cells, and macrophages, indicating an immunosuppressive TME. **(B)** Correlation analyses between risk scores and various TME characteristics are presented as scatter plots, showing significant negative correlations between risk scores and both immune scores (r = −0.57, q = 0) and stromal scores (r = −0.41, q = 0), along with a positive correlation between risk scores and tumor purity (r = 0.56, q = 0). These findings suggest that higher risk scores are associated with lower immune infiltration and stromal content, reflecting a more tumor-promoting microenvironment.

To investigate the relationship between our prognostic model and the tumor microenvironment (TME), we performed an immune infiltration analysis. As shown in Figure 4A, a heatmap displays the expression levels of various immune cell types across high-risk and low-risk groups. The patterns of immune cell infiltration indicate a distinct immune landscape, with significant differences observed between the two risk categories. High-risk patients exhibit a lower abundance of several immune cell types, including CD4^+^ T cells, regulatory T cells, and macrophages, compared to their low-risk counterparts.

Furthermore, we conducted correlation analyses to explore the associations between the risk scores derived from our model and various TME characteristics, including stromal score, immune score, ESTIMATE score, and tumor purity. Figure 4B presents scatter plots illustrating these relationships, with significant negative correlations observed between risk scores and both immune scores (r = −0.57, q = 0) and stromal scores (r = −0.41, q = 0). Additionally, a positive correlation was noted between risk scores and tumor purity (r = 0.56, q = 0). These findings indicate that higher risk scores correspond to lower immune infiltration and stromal content, reflecting a more tumor-promoting microenvironment.

In summary, our analysis underscores the interplay between the prognostic risk model and TME characteristics, highlighting the role of immune infiltration in influencing patient prognosis. The results suggest that a less immune-active and more tumor-dominant microenvironment is associated with poorer outcomes in patients with higher risk scores.

### Prognostic model and clinical significance of TBC1D16 in skin melanoma

**FIGURE 5:**
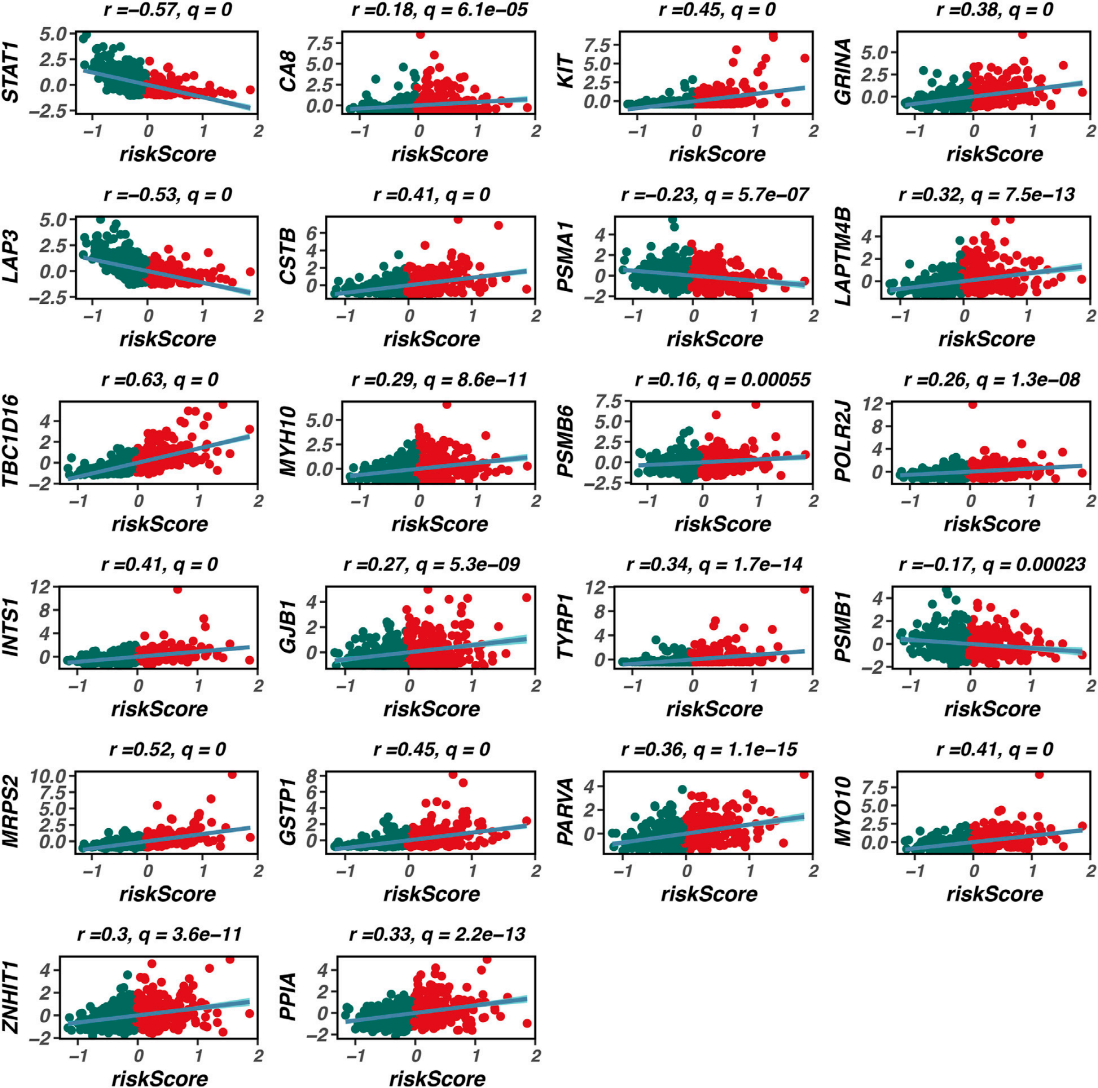
Composition of the prognostic model. This figure illustrates the composition of the prognostic model, consisting of 22 genes. Among these genes, TBC1D16 shows the strongest positive correlation with the model’s risk score, indicating its significant association with poor prognosis in patients with cutaneous melanoma. The construction of this model highlights the importance of these genes in predicting patient outcomes in melanoma.

As illustrated in Figure 5, our prognostic model is composed of 22 genes. Among these, TBC1D16 exhibits the strongest positive correlation with the model’s risk score, indicating that it is most closely associated with poor prognosis in patients with skin melanoma. To further explore the clinical significance of TBC1D16, we conducted an analysis using the BEST database.

**FIGURE 6:**
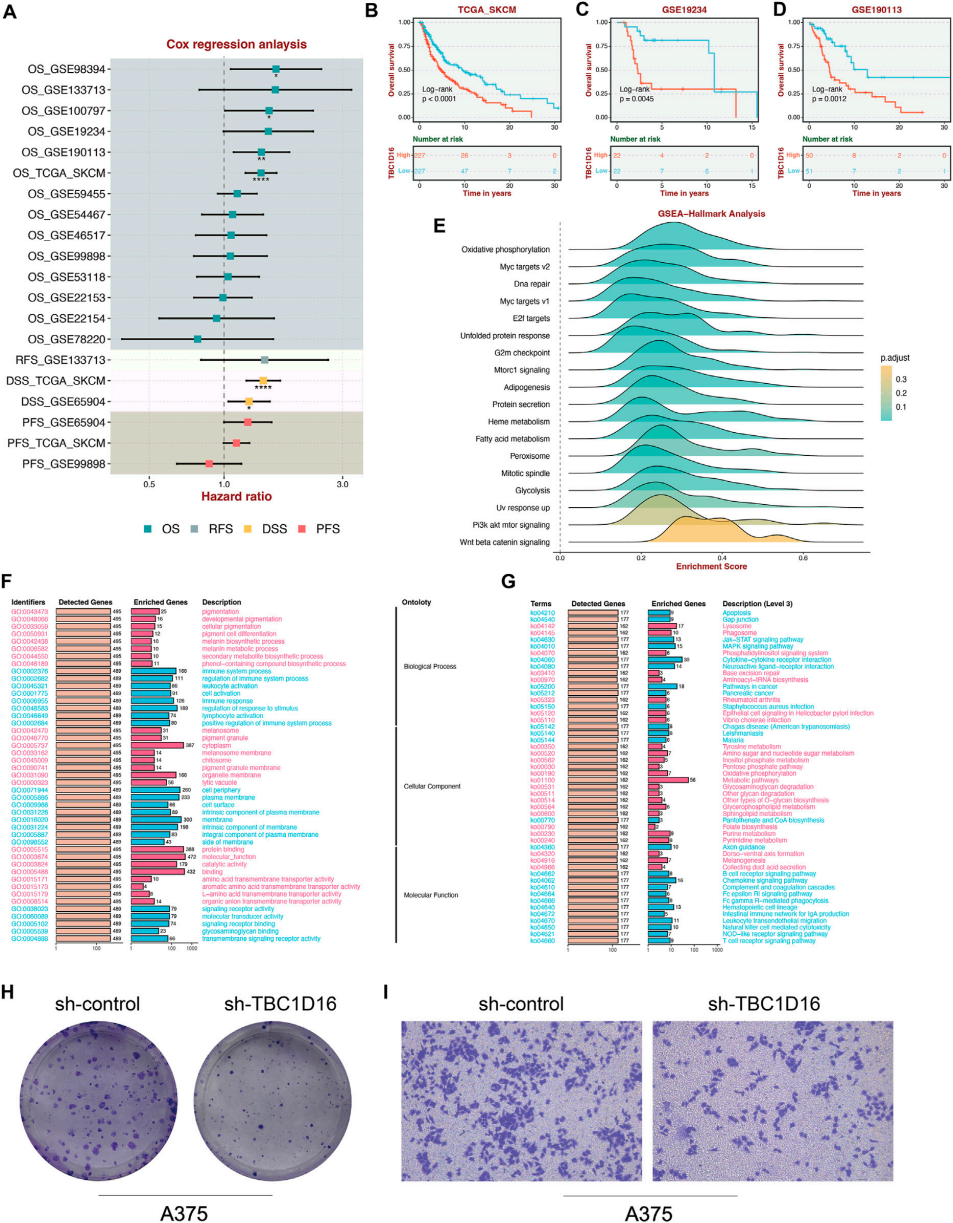
Validation of key gene TBC1D16 in skin melanoma. This figure presents the validation results for the key gene TBC1D16. Cox regression analysis revealed that TBC1D16 is significantly associated with unfavorable prognosis across multiple cohorts **(A)**. Kaplan-Meier survival curves demonstrate that high expression levels of TBC1D16 correlate with worse patient outcomes in the TCGA, GSE19234, and GSE190113 datasets **(B–D)**. Gene Set Enrichment Analysis (GSEA) further indicates that TBC1D16 is highly correlated with several cancer-related pathways, including oxidative phosphorylation and Myc target pathways **(E)**. Gene Ontology (GO) and Kyoto Encyclopedia of Genes and Genomes (KEGG) enrichment analyses reveal that TBC1D16 is associated with various cancer functions and pathways **(F, G)**. Finally, we validated the functional role of TBC1D16 in the A375 cell line, showing that knockdown of TBC1D16 resulted in a significant decrease in both proliferation and migration capabilities of skin melanoma cells **(H, I)**.

Cox regression analysis revealed that TBC1D16 is consistently associated with unfavorable prognosis across multiple cohorts (Figure 6A). Additionally, Kaplan-Meier (K-M) survival curves demonstrated that high expression levels of TBC1D16 correlate with worse patient outcomes in the TCGA, GSE19234, and GSE190113 datasets (Figures 6B–D). Gene Set Enrichment Analysis (GSEA) further indicated that TBC1D16 is highly correlated with several cancer-related pathways, including oxidative phosphorylation and Myc target pathways (Figure 6E). Furthermore, Gene Ontology (GO) and Kyoto Encyclopedia of Genes and Genomes (KEGG) enrichment analyses revealed that TBC1D16 is associated with various cancer functions and pathways (Figures 6F, G). Finally, we validated the functional role of TBC1D16 in the A375 cell line. Our experiments demonstrated that knockdown of TBC1D16 resulted in a significant decrease in both proliferation and migration capabilities of skin melanoma cells (Figures 6H, I).

## Discussion

Our study presents significant insights into the role of deubiquitinating enzymes (DUBs) in cutaneous melanoma, revealing their potential as novel biomarkers and therapeutic targets. The alarming rise in melanoma incidence underscores the urgency for effective prognostic tools and treatment strategies. Our analysis of single-cell RNA sequencing data highlighted the heterogeneity of melanoma, with distinct cellular populations exhibiting varying deubiquitination activities. This finding aligns with previous studies suggesting that DUBs modulate critical signaling pathways that influence tumor progression and metastasis (Feng et al., 2021).

The construction of our prognostic model using ensemble machine learning algorithms represents a noteworthy advancement in melanoma research. By integrating differential expression data with correlation analyses, we identified a robust set of biomarkers that accurately stratify patients into high-risk and low-risk categories. The model’s high concordance index across multiple cohorts indicates its potential clinical applicability, offering a reliable tool for guiding therapeutic decision-making.

Our investigation into immune cell infiltration revealed notable differences in the tumor microenvironment between risk groups, emphasizing the intricate interplay between DUB activity and immune modulation. The immune landscape of tumors is critical for understanding patient outcomes, and our results suggest that deubiquitination processes may influence immune evasion mechanisms in melanoma. Future studies should explore the precise mechanisms by which DUBs alter immune responses, as targeting these pathways could enhance the effectiveness of immunotherapies.

Furthermore, our functional assays targeting TBC1D16 provide compelling evidence for its role in melanoma cell proliferation and migration. TBC1D16 (TBC1 Domain Family Member 16) is a protein involved in the regulation of intracellular vesicle trafficking (Vizoso et al., 2015). In cancer, particularly melanoma, TBC1D16 has been associated with tumor progression due to its role in promoting cell proliferation, migration, and invasion. Elevated TBC1D16 expression has been linked to poor prognosis in melanoma patients, suggesting its potential as a prognostic biomarker. Studies indicate that silencing TBC1D16 can reduce melanoma cell migration and proliferation, highlighting its significance in tumor aggressiveness and as a potential therapeutic target. This gene’s involvement in key cellular processes may offer a promising avenue for therapeutic intervention. By silencing TBC1D16, we demonstrated a significant reduction in the migratory potential of melanoma cells, indicating its potential as a target for novel therapies aimed at limiting metastasis.

In conclusion, our findings establish a crucial link between deubiquitinating enzyme (DUB) activity and melanoma progression, emphasizing the need for continued exploration of deubiquitination pathways in cancer biology. The prognostic model developed in this study offers a promising tool for stratifying melanoma patients based on risk, which could improve clinical decision-making and treatment personalization. However, while the model demonstrates strong predictive performance in multiple cohorts, several challenges remain for its clinical implementation. For instance, the integration of this model into routine clinical practice requires further validation in larger, independent patient cohorts to confirm its robustness and generalizability. Additionally, the clinical utility of targeting DUBs as therapeutic interventions in melanoma needs to be carefully evaluated through clinical trials, as altering DUB activity could have unintended consequences on immune response or tumor microenvironment dynamics. Further investigations are essential to explore the optimal strategies for targeting these pathways without compromising patient safety. Moreover, the biological variability across different patient populations, including tumor heterogeneity and immune landscape differences, may affect the model’s predictive accuracy. Therefore, refining the model to account for these factors will be critical in enhancing its clinical applicability. In summary, while this study provides a solid foundation for developing DUB-related prognostic biomarkers and therapies, future research should focus on addressing these challenges to ensure that such models can be effectively utilized in clinical settings.

## Data Availability

The original contributions presented in the study are included in the article/Supplementary Material, further inquiries can be directed to the corresponding author.
