# Supplementary material for: Exploring the impact of deubiquitination on melanoma prognosis through single-cell RNA sequencing
Source: Front Genet. 2024 Dec 5;15:1509049. doi: 10.3389/fgene.2024.1509049 (PMC11659643; doi:10.3389/fgene.2024.1509049)
Supplement: Supplementary file 1 [file Table1.docx]

| **Oligonucleotides** | **Nucleotide sequence (5'-3')** |
| --- | --- |
| **shRNA** |  |
| Scramble control | GCUUCGCGCCGUAGUCUUA |
| sh-TBC1D16 | GTGGAAATACTGCACCGAGAT |

**Table S1. Oligonucleotides used in research**
